# Feasibility of Implementing Evidence-based Practices for Suicidality Management in the Emergency Department

**DOI:** 10.5811/westjem.50581

**Published:** 2026-03-13

**Authors:** Ashlyn Burns, Lauren O’Reilly, Elizabeth Linhart-Espino, Katherine LeFevre, Zachary Adams, Rachel Yoder, Paul Musey, Casey Pederson

**Affiliations:** * Indiana University School of Medicine, Department of Psychiatry, Indianapolis, Indiana; † Indiana University School of Medicine, Department of Pediatrics, Indianapolis, Indiana; ‡ Indiana University School of Medicine, Department of Emergency Medicine, Indianapolis, Indiana

## Abstract

**Introduction:**

Best practice recommendations and guidelines for the assessment and management of suicidality within the emergency department (ED) have recently been updated. Despite national efforts to improve the management of suicidality in the ED, evidence-based practices remain underused with varied uptake among EDs and clinical team members. Given that the ED is a common point of entry for many people with suicidality, implementation of evidence-based strategies are needed to increase access to these strategies and improve patient outcomes.

**Methods:**

To generate insights about the feasibility of implementing evidence-based practices for suicidality management, we developed a semi-structured interview guide focused on factors expected to influence the implementation process using a novel application of the Organizational Readiness for Innovation Implementation Framework. Working from a list or 80 EDs in the state of Indiana, we recruited emergency physicians, nurses, physician assistants, and social workers to participate in interviews. Interviews lasted approximately 45–60 minutes and were recorded, transcribed, and qualitatively analyzed using a multistage thematic analysis process.

**Results:**

We conducted 11 interviews with ED clinical team members from eight EDs in Indiana, representing 10% of the 80 EDs invited to participate in our study. Identified barriers to effective implementation included a general lack of resources, resistance to change among clinical team members, and competing demands in the ED setting. Facilitators included openness to attending training, openness to implementing change in the ED, and leadership support. Openness to change and commitment to change appeared to be driven by discontent with current processes and a desire to improve patient experiences.

**Conclusion:**

Considering mixed attitudes toward suicidality management and questions about whether these services are within the scope of clinicians who work in the ED, efforts to increase uptake of evidence-based practices may involve a multifaceted approach that involves identifying and training team members who are open and ready for change, while simultaneously establishing stronger relationships between ED clinical team members and behavioral health clinicians with specialized training who can provide consultative services in the ED.

## INTRODUCTION

Suicide is a growing national health emergency in the United States (US). As of 2023, suicide was the 11^th^ leading cause of death in the US, with over 49,000 deaths by suicide.^1^ When asked about the past year, nearly 13 million adults reported suicidal ideation, four million reported having a suicide plan, and 1.5 million reported attempting suicide.^2^ The emergency department (ED) is an important part of the care continuum for suicidal crises.^3^–^6^ Between 2008–2020, ED visits for suicidality (ie, suicidal ideation, suicidal intent, suicide planning, and suicide attempts) increased among all age groups.^3^,^4^ As a unique and integral setting within healthcare systems that provides 24/7 care to anyone in need, regardless of condition, background, or status,^7^ EDs are often the first point of contact for people seeking suicide-related care. Emergency clinicians may assess suicide risk, provide acute stabilization, and connect people to appropriate follow-up care. Numerous studies have shown that most people who died by suicide had at least one healthcare visit in the year prior to suicide, typically in an outpatient or ED setting.^3^–^6^ More specifically, one retrospective review of people who died by suicide after an ED visit found that only 50% were identified as having a mental health need during their visit, indicating gaps in screening.^5^

Furthermore, while one-third of all ED visits are primarily paid by Medicaid or disability-related Medicare, more than half of all ED visits for suicidality are covered by these payors.^6^ Compared to people with private insurance, people with Medicaid or disability-related Medicare are less likely to have access to mental health services due to social and economic barriers and more likely to have comorbid psychiatric disorders,^6^ which may contribute to the disproportionately high rate of ED visits for suicidality among this population. Thus, there is an urgent need to understand how emergency clinicians can most effectively manage suicidality, especially when it comes to the most vulnerable people who are not otherwise accessing or engaging in care.

Best practice recommendations and guidelines for the assessment and management of suicidality within the ED have been updated in the past several years.^8^–^12^ In 2020, the American College of Emergency Physicians recommended the following evidence-based practices (in addition to stabilization and assessment for environmental safety): suicide screening; suicide risk assessment; safety planning (eg, lethal means counseling); and discharge planning (eg, inpatient and outpatient care).^10^ The Health Resources and Services Administration and Joint Commission also recommend universal suicide screening.^8^,^9^

To improve hospital performance and increase accountability for follow-up care, the National Committee for Quality Assurance has established quality measures focused on the receipt of follow-up care after an ED visit related to self-harm.^13^,^14^ Yet despite national efforts to improve the management of suicidality in the ED, evidence-based practices remain underused with varied uptake among EDs and clinical team members.^4^,^15^ In one multisite study across seven states implementing universal suicide screening, only 36% of nurses and 8% of physicians reported performing suicide screening at baseline.^15^ Another study estimated that only 16% of patients with suicidal presentations received an assessment from a mental health professional at their point of contact.^4^ Even when evidence-based practices for suicidality are adopted, the extent to which they are implemented with high fidelity is unclear.^16^

**Population Health Research Capsule**

What do we already know about this issue?

*Despite national efforts to improve the management of suicidality in the emergency department (ED), evidence-based practices remain underused*.

What was the research question?

*What factors might influence the implementation of evidence-based practices for managing suicidality in the ED?*

What was the major finding of the study?

*Limited resources, resistance to change, and competing demands may act as barriers to evidence-based suicidality management*.

How does this improve population health?

*Organizational leaders looking to improve care and reduce deaths by suicide can use our findings to develop strategies for overcoming implementation barriers*.

While implementation science frameworks have been used by researchers to review barriers and facilitators to suicide prevention services,^17^ this research was not specific to the ED setting. Research examining the implementation of evidence-based practices for suicidality in the ED has primarily been limited to suicide screening. This work has revealed numerous barriers to suicide screening in EDs (eg, limited time, limited privacy, limited mental health training, skepticism or discomfort about evidence-based practices for suicidality, and prioritization of physical health emergencies) and minimal standardization in the management of suicidality, especially post-screening. Indeed, our understanding of barriers and facilitators to implementation of post-screening evidence-based practices for suicidality (eg, assessments, brief interventions, referrals, and follow-up) in the ED remains limited, inhibiting the development of effective strategies for encouraging consistent, committed use of these practices in the ED.

While enhanced upstream prevention and mental health services may decrease ED utilization,^18^–^20^ given that the ED is a common point of entry for many people with suicidality, additional research is needed to inform the development of implementation strategies to increase the accessibility of evidence-based practices and improve patient outcomes. Thus, our goal in this study was to assess the feasibility of implementing evidence-based practices for suicidality in the ED. We conducted semi-structured qualitative interviews with ED clinical team members throughout Indiana who were recruited as part of a broader initiative to understand factors influencing the adoption and implementation of evidence-based practices for managing suicidality in emergency settings. Findings from our study offer insights into potential barriers and facilitators of implementation, which can help inform the development of strategies to disseminate these practices for suicidality management in the ED and increase access to life-saving interventions.

## METHODS

### Interview Guide Development and Theoretical Framework

We developed a semi-structured interview guide to collect information about current practices for suicidality management in the ED and factors that may influence the implementation of evidence-based practices (ie, innovations for suicidality management). All interview questions were designed to generate insights into factors expected to influence implementation feasibility, including organizational readiness and determinants of implementation effectiveness (Figure). We adapted the constructs in the Figure by applying constructs originally proposed by the Weiner (2009) theory of organizational readiness and Klein and Sorra’s (1996) theory of innovation implementation to the context of implementing evidence-based practices for suicidality management.^22^–^25^ Given our primary outcome of implementation feasibility, we specifically tailored our guiding framework to focus on constructs related to implementation effectiveness that can be assessed pre-implementation. Based on past research,^15^–^19^ we anticipated certain topics such as limited training, discomfort with managing suicidality, competing demands, and limited discharge options to be raised—topics that would be reflected in themes aligned with this framework.^22^–^25^

We define implementation effectiveness as the consistency, quality, and appropriateness of an organization’s use of evidence-based practices for suicidality management. The four theoretical constructs expected to influence implementation effectiveness are organizational readiness for change, implementation policies and practices, implementation climate, and innovation-values fit.^22^–^25^ Organizational readiness for change is the extent to which employees are prepared to make changes in organizational policies and practices needed to implement evidence-based practices for suicidality management. Implementation policies and practices include plans, activities, structures, and strategies that may influence the implementation of evidence-based practices for suicidality management. Implementation climate refers to perceived rewards, supports, and expectations for suicidality management. Finally, innovation-values fit refers to the alignment of evidence-based practices for suicidality management with ED clinical team members’ individual values.

### Participant Recruitment and Interview Procedures

To facilitate recruitment of ED clinical team members, we compiled a list of EDs in Indiana and points of contact by searching online directories (eg, the Indiana Rural Hospital Association) and individual hospital websites. We sent email requests to the leadership of 80 identified EDs and invited them to share our study information with all ED clinical team members. Since we were interested in capturing perspectives from team members with various roles in the ED, we defined ED clinical team members to include anyone delivering direct patient care in the ED setting (eg, physicians, physician assistants, nurses, social workers, etc). To encourage participation, we sent two follow-up emails to all non-respondents. All participants were offered a $50 gift card as an incentive. Interviews lasted approximately 45–60 minutes and were conducted, recorded, and transcribed using Microsoft Teams (Microsoft Corporation, Redmond, WA). After all interviews were completed, the transcripts were reviewed, cleaned for accuracy, and de-identified. We then uploaded the clean, de-identified transcripts into NVivo 14 (Lumivero, Denver, CO) for analysis. This study was approved by the Institutional Review Board at Indiana University (IRB: #18122).

### Qualitative Coding and Thematic Analysis

We qualitatively analyzed all interviews using a multistage process similar to that used in prior implementation studies.^24^–^26^ In the first phase of our analysis, we used a deductive, binned coding approach in which we reviewed all transcripts for the presence of quotes related to any of the theoretical constructs in our codebook (eg, organizational readiness for change, implementation policies and practices, etc).^27^,^28^ In addition to categorizing quotes by theoretical construct, we also differentiated whether each coded quote referred to a potential facilitator of or barrier to innovation implementation.^24^–^26^ To ensure agreement between coders and test our coding scheme, five members of the research team independently coded the same transcript.

The research team met as a group to review this first transcript, compare coding, and refine the coding framework by clarifying definitions of codes as needed. The remaining transcripts were then divided up with at least two members of the research team assigned to each transcript. During this phase of the coding process, the research team met on a weekly basis to review agreement between coders. In cases where two coders applied different codes to the same excerpt, we discussed these disagreements in depth until a consensus was reached.^24^–^26^ The research team reviewed and resolved discrepancies on all transcripts, allowing us to achieve 100% agreement across all codes (kappa = 1.0).

After reaching agreement on all binned codes applied to the full set of transcripts, we conducted a second, inductive analysis on all previously coded excerpts. In this phase, two members of the research team independently reviewed all excerpts within each theoretical construct to uncover deeper insights. In doing so, each researcher generated a list of emergent themes, and then the researchers met to discuss emergent themes and reconcile them into a single list. Finally, to ensure face validity of all identified themes, the research team met to review the reconciled lists of themes and illustrative quotes as a group.

## RESULTS

### Sample

We conducted 11 interviews with ED clinical team members from eight different EDs in the state of Indiana, representing 10% of the 80 total EDs invited to participate in our study. In terms of organizational operation, six (75%) of the EDs represented by our study participants were private nonprofit hospitals and two (25%) were public nonprofit hospitals. In terms of geographic area, three (37.5%) EDs were located in metropolitan counties, three (37.5%) in suburban counties, and two (25%) in rural counties. Additionally, two (25%) EDs were designated as critical access hospitals. As for characteristics of individual clinical team members, our sample included five nurses (45.5%), three physicians (27.3%), two physician assistants (18.2%), and one social worker (9.1%). In terms of education levels, our sample included participants with a mix of bachelors-, masters-, and doctoral-level training. At the time of the interviews, all participants were actively providing clinical care in an ED setting at least part time and two participants were simultaneously holding an administrative position. Below, we summarize emergent themes within each theoretical construct of the Organizational Readiness for Innovation Implementation Framework.

### Organizational Readiness for Change

Three themes emerged within the organizational readiness for change construct: resource availability; change efficacy; and change commitment (Table 1). Within the resource availability theme, barriers focused on a lack of resources, while facilitators focused on resources already in place. Within the change efficacy theme, barriers focused on uncertainty about implementing change, while facilitators focused on beliefs that change is possible. Within the change commitment theme, barriers focused on resistance to change while facilitators focused on a desire for change.

### Implementation Policies and Practices

Three themes emerged within the implementation policies and practices construct: training; existing policies; and communication structures (Table 2). Within the training theme, barriers focused on challenges to training participation while facilitators focused on training already in place. Within the existing policies theme, barriers focused on policies that inhibit suicidality management while facilitators focused on supportive policies. Within the communication structures theme, barriers focused on gaps in communication while facilitators focused on established communication channels.

### Implementation Climate

Three themes emerged within the implementation climate construct: scope of services; behavioral health saliency; and leadership support (Table 3). Within the scope of services theme, barriers focused on suicidality management being seen as out of scope while facilitators focused on suicidality management being seen as an organizational priority. Within the behavioral health saliency theme, barriers focused on limited awareness of suicidality management while facilitators focused on increased awareness. Within the leadership support theme, barriers focused on leadership not being viewed as supportive of suicidality management while facilitators focused on views that leadership are supportive of / receptive to improving suicidality management.

### Innovation-Values Fit

Two themes emerged within the innovation-values fit construct: individual values and individual attitudes (Table 4). Within the individual values theme, barriers focused on being satisfied with current processes while facilitators focused on a desire for better processes. Within the individual attitudes theme, barriers focused on stigmatizing views about suicidality management while facilitators focused on perceived intrinsic value of suicidality management.

## DISCUSSION

The purpose of this study was to assess the feasibility of implementing evidence-based practices for suicidality management within the ED. We conducted qualitative interviews with ED clinical team members using an interview guide designed to assess determinants of ED readiness to implement evidence-based practices for suicidality,^22^–^25^ and we then categorized emerging themes within each conceptual domain as a barrier or facilitator. Barriers that may be particularly challenging to overcome include a general lack of resources, resistance to change among clinical team members, and competing demands in the ED setting. Given that resource constraints and competing demands may be outside the ED’s control, we recommend that anyone looking to promote the uptake of evidence-based practices for suicidality management begin by developing strategies for overcoming resistance to change among clinical team members. While additional research is needed to test specific strategies for overcoming resistance to change in the ED setting, those looking to develop such strategies may draw on insights generated by prior studies in other healthcare contexts. For example, persistent and ongoing communication has demonstrated effectiveness in changing team dynamics and processes.^29^ Thus, future research should assess whether persistent and ongoing communication about the importance of evidence-based practices for suicidality management improve the uptake of EBPs over time.

Unsurprisingly, subthemes related to organizational readiness for change were the most prominent, centering on limited resources both within (eg, limited staff, space) and outside (eg, lack of short-term hospitalization options) the ED. Consistent with prior research,^17^ there is a clear need for additional resources to support successful implementation of evidence-based practices for suicidality management, including both ED staff trained in screening for suicidality and community resources to provide follow-up care for patients who screen positive for suicidality.^11^ While nearly all participants discussed a lack of resources as a barrier, one individual from an urban ED noted available resources as a facilitator to implementation, and described how existing training infrastructure (eg, availability of continuing education) could serve as a resource to support the implementation of evidence-based practices.

Considering the lack of perceived self-efficacy toward suicidality management and desire for more training expressed by clinical team members, increasing participation in training may be an important strategy for increasing uptake of evidence-based practices. However, this will require overcoming negative attitudes toward training, which also emerged as a subtheme. Importantly, providing training in evidence-based practicess alone may not result in consistent, committed use of these practices.^30^ Therefore, multipronged strategies that go beyond training are needed are needed to simultaneously address other implementation barriers. More specifically, additional barriers to change commitment centered on a lack of incentives or recognized need to address mental health concerns among patients within the ED. Thus, strategies for increasing individual motivation may be needed, especially in the absence of institutional resources.

Facilitators and barriers related to formal policies and communication structures focused on existing policies that mirrored each other. For example, existing policies that participants reported as facilitators included standardized suicide screening protocols, risk stratification and triage policies, community resource guides, and safety policies (eg, room sitters) in place. On the other hand, participants from EDs where such policies are either lacking or poorly implemented described the lack of formal protocols for suicidality management as a barrier. Notably, recent policy changes related to emergency detention orders in Indiana were frequently discussed as both a facilitator and a barrier. Clinical team members described the ability to keep patients for 48 hours without judicial approval as helpful for ensuring patient safety; however, clinical team members also expressed concerns about infringing on patient rights by enacting this process, causing patients distress, and perhaps adding to medical distrust. Future research should further examine the role of emergency detention orders, as well as other external policies governing suicidality management in the ED.

Within the implementation climate and innovation-values fit domains, we identified a critical mismatch between the perceived scope of services offered through the ED, personal sense of responsibility for addressing suicide risk, and how patients and other clinical team members use the ED when suicide risk arises. While some clinical team members identified suicidality management as within the scope of the ED and supported by institutional leadership, others clearly indicated that suicidality was not and, perhaps, should not be prioritized within the ED. These clinical team members tended to focus on the competing demand for services within the ED, highlighting that patients experiencing suicidal thoughts and behavior are often “competing” for the attention of limited staff treating patients experiencing emergent issues such as cardiac events, gun violence, broken bones, and a myriad of other “physical health” concerns.

The fast-paced nature of the ED may contribute to stigmatizing beliefs that suicidal patients “take advantage” of ED resources as well as individual beliefs that the ED is already appropriately serving patients experiencing suicidal crises through stabilization.^31^ Many emergency clinicians felt that they were already doing enough by providing emergency stabilization and that provision of other services for those experiencing suicidal thoughts and behavior (eg, initiating brief interventions, coordinating follow-up care) was outside the ED’s scope. However, given that patients who present to the ED with suicidal ideation are at highest risk during the days following discharge from the ED, the coordination of follow-up care is essential for preventing loss of life.^11^ Guidance for improving access to comprehensive mental health treatment reinforces the importance of providing guided referrals from the ED and connecting individuals directly to follow-up resources to ensure continuity of care.^9^

Understandably, clinical team members may be wary of practicing beyond their scope by initiating brief interventions or treatments in the ED, which raises questions about whether people experiencing suicidal thoughts and behavior would be better served through other treatment delivery models and resources outside of the ED. In addition to seeking to improve access to evidence-based practices in the ED, future research should also explore strategies for shifting the public health response to suicidal thoughts and behavior to ensure rapid connection to life-saving treatment.

## LIMITATIONS

This study has limitations. We were able to interview clinical team members representing eight EDs in Indiana, providing insight into current barriers and facilitators to the management of suicidality across multiple settings, including both rural and urban communities. We were also able to capture perspectives from diverse clinical team member types. However, our findings represent individual perspectives only, which may not necessarily be reflective of formal processes and procedures in place if people are not aware of them. Further, the provision of incentives may have introduced participation bias. Consequently, the conclusions of this study may not be widely generalizable.

## CONCLUSION

Considering mixed attitudes toward suicidality management and questions about whether these services are within the scope of ED clinical team members in the ED, efforts to increase uptake of evidence-based practices may consider a multifaceted approach that involves identifying and training ED clinical team members who are open and ready for change, while simultaneously establishing stronger relationships between them and behavioral health clinicians with specialized training who can provide consultative services in the ED. Such an approach also requires increasing structures and resources within ED settings to practically facilitate change. Formalizing communication structures, embedding behavioral health clinicians into the ED workflow, and/or increasing access to other consultation options and opportunities may improve and sustain implementation of evidence-based practices for suicidality management within the ED.

## Figures and Tables

**Figure:**
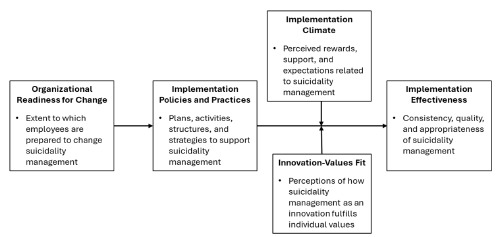
Determinants of emergency department readiness to implement evidence-based practices for suicidality management.

**Table 1:** Organizational readiness for change: the extent to which employees are prepared to change suicidality management. Subthemes that emerged in a study assessing determinants of readiness to implement evidence-based practices for suicidality in the emergency department.

**Subtheme:** Resource Availability	

**Barriers:** Lack of resources	**Facilitators:** Resources in place
**Limited ED staff:** “I honestly think it would just be staffing, just getting the right people hired. Just because, in general, I think places are just struggling to get people.” **Limited psychiatric staff:** “We just have had such an increased volume of patients with mental health crises that you know it’s hard to keep up. Sometimes, we might have 6, 8, 10 patients who need an evaluation by the psychiatric team and it doesn’t happen quickly because they’re basically waiting in line.” **Limited time:** “There’s a lot of opportunity for education. But I think the problem is time and ability and everybody is stretched thin to begin with. So how do you continue to teach that and keep people engaged?” **Limited funding:** “I think just having the resources, I think that’s always the biggest thing is where’s the money going to come from? How are we going to staff it? When is the busiest hours? I think they would support it if we had the funds and the ability to support it and the team. **Lack of private space:** “The way our ED is set up our psych rooms are in the middle of other rooms. So, I think it’s very challenging because it’s such a stimulating environment. I know some other EDs have a separate area that’s away from everything else.” **Boarding due to lack of placement:** “I think some sort of expanded facility or short-term inpatient stay until they can get placement would be helpful. Especially for pediatrics, like sometimes we will have pediatric patients who need an inpatient psychiatric facility, and it can take days to get them placed. So, then they’re stuck in a room [in the ED] and getting really no treatment. So, you know, placement is really a big issue.”	**Existing training offered through organization:** “We do like yearly learning e-learning on suicide risk and things like that.” **Continuing education credits:** “If they’re getting continuing education credits for it, that may also help.” **Perceived lack of barriers:** “I feel like if we had training that we could then come and take back and give our providers, I think they would be like, oh, that’s helpful. So, I don’t see any barriers from that standpoint.”

**Subtheme:** Change Efficacy	

**Barriers:** Uncertainty about implementing change	**Facilitators:** Belief that change is possible
**Lack of perceived competency:** “I don’t think we’re equipped at all to truly handle that kind of stuff.” **Lack of knowledge about mental health resources:** “I didn’t even know who our social work team was until about a month ago when they came down and introduced themselves and they were like, “You have a psych patient, do you want me to go talk to them about the process?” I was like, “Yes.” Because I don’t even know the process of what happens when you get there. So, I think that’s the scary part too, is what are our resources? Because like I said, I don’t even know what our resources are.” **Uncertainty about evidence-based practices for suicidality management /suicide prevention:** “I think more current updates or current literature, like what evidence-based evaluation or what tools are most proven as far as evaluating suicide or the likelihood of suicide. What are the tools that are, you know, most relevant to prevention. What options do patients have?”	**Self-efficacy:** “It’s talked about more often than like, you know, when it was years ago on the medical floor when [suicide risk] was something that nurses and staff had to assess, that was all new. But I think it, you know, they tend to be more comfortable with it now.” **Openness to collaboration:** “I definitely think there could be way more services offered down in our ED because it’s right now like [our behavioral health team] makes the decision. It would be kind of nice if we had more behavioral health people able to come speak with them and chat with them and get a plan going or someone who can set them up outpatient.” **Openness to innovation:** “We’re open [to change]. You always have to be careful. You know, it depends on how resource intensive it is… but we have certainly, we certainly have a history of implementing such things.” **Psychological safety with leadership:** “I feel like when there is needed to be change, I feel very comfortable actually going to my leaders and suggesting a change and if it’s in regards to patient care, they are very willing to do what it takes to make it happen for to do what’s best for the patient.” **Compassionate clinicians:** “I feel like our physicians are really good at dealing with people in a crisis. They’re just very kind and caring, and I think that’s really helpful.” **Foundational knowledge from prior training:** “Medical school and residency cover a lot of issues related to safety, de-escalation, and suicidal ideation and all of that.” **Desire for change to improve the patient experience:** “I wish that we had a separate area we could put people who were coming in for suicidal ideation or mental health crises so that it wasn’t as embarrassing. This is not ideal, but sometimes these patients are in the hallway because there’s not a room available just so we could observe them closely... I wish that there was a separate area with maybe even a separate team that could really take the time to make these people feel human still and respected.”

**Subtheme:** Change Commitment	

**Barriers:** Resistance to change	**Facilitators:** Desire for change
**Resistance to change/lack of incentives:** “I think it’s going to be difficult to get people to do the training unless there’s some sort of incentive behind it. And also I think people kind of like already are kind of set in how they manage these things, so I think that they might be a little resistant to it from that standpoint.” **Closed-mindedness about the need to address mental health concerns:** “Mental health is everywhere, and I sometimes think people are closed-minded into accepting that. I think people are like oh, I don’t need that class I know how to deal with it. That’s why we have medicine.” **Lack of recognized need:** “I think we’re pretty set with what we have in terms of having behavioral health consults.”	**Desire for more behavioral health resources/dedicated staff:** “It would be kind of nice if we had more behavioral health people able to come speak with them and chat with them and get a plan going or someone who can set them up outpatient. More people who are trained in therapeutic communication. I wish we could bring in actual nurses that are trained in psychiatric care and mental health and doctors specifically.” **Desire for training on therapeutic communication and suicide prevention:** “We’ve had therapeutic communication classes. However, I definitely don’t think it’s done enough. I wish we had them more often. It’s more so in the beginning like when you’re on orientation in the first, like, six months. However, I wish it was continued like every six months to kind of touch up upon that especially therapeutic communication. For sure, suicide prevention, yes, I wish we definitely had more of that.” **Desire for training on prescription medications:** “I think it would be nice to better understand... we get a lot of, “Hey, I’m struggling. I can’t get into a mental health provider because it’s booked and we don’t have enough people, so it may be three months before I can get in. I would like to be started on medications.” Since I’ve been in school, I can recognize psychiatric medications... it’d be nice to have a hey, these are a couple drugs that you can start to get the patients through until they can get into someone because a lot of times, if they’re not having active suicidal ideations, it’s people coming in desperate for something. They’re like, I just need help until I can get here.” **Desire to collaborate with behavioral health professionals:** “I think our behavior health team could come and chat with us honestly and like have a conversation of like what it looks like because once we send them over they are off somewhere else like we don’t really see them again... so it would be kind of nice to hear from them to see what happens after we send them over there, what their process is, and how we can work with them to make it better.”

*ED*, emergency department.

**Table 2:** Implementation policies and practices: plans, activities, structures, and strategies to support suicidality management.

**Subtheme:** Training	

**Barriers:** Challenges to training participation	**Facilitators:** Training already in place
**Reliance on professionals with specialized training:** “If we had like something to follow up that form that we asked everyone like something standardized to see what resources would best help them, I think that would be really helpful because I think a lot of the time it’s like provider discretion or relying really heavily on like social work to provide them resources.” **Existing training not viewed as helpful or applicable:** “We maybe take a class on de-escalation or when they’re in orientation, they’re taught what the policies and procedures are behind it. But until it actually is happening, it’s hard to conceptualize what’s going to happen or what might work for each individual patient.” **Training viewed as potentially redundant:** “Is it training that’s relevant or is it training that’s sort of redundant based on my practice board certifications?”	**Existence of training modules/platform:** “We do yearly learning, e-learning on suicide risk and things like that.” **Availability of training credit:** “If they’re getting continuing education credits for it, that may also help.” **Opportunities for debriefing after crisis incidents:** “I just feel like there should be more talk about it. Like, if there is a situation that maybe doesn’t go so well, debriefing, talking about what we could do better. A lot of times we do those with the more serious things, you know, a cardiac arrest or something like that we will debrief after, but I don’t think we do that, I’ve never done that actually, after a bad incident with someone who is experiencing a crisis... I’ve never had a debrief and I think that would be helpful in making change because that just gets you talking about what went well and not so well.” **Lack of perceived barriers to training:** “There’s not necessarily any policies in place that prevents it from happening.”

**Subtheme:** Existing Policies	

**Barriers:** Policies that inhibit suicidality management	**Facilitators:** Policies that support suicidality management
**Lack of formalized protocols:** “We have the doctor’s chat with them, we have our behavioral team people chat with them and kind of get a plan of care going. We keep a close eye on them. It’s kind of, there’s not a super great plan for that to be honest... they just kind of use your own judgment and the doctor’s judgment to see kind of how you want to go from there.” **Differences in insurance coverage:** “That sustaining part is always like trying to figure out, well, how are we going to do this? How much time? How much spending? Like peer recovery now it’s you can bill Medicaid, but then my question is, okay, you can bill Medicaid. What if someone that has commercial insurance needs that service, do they not get billed? You know and then how’s that fair to the person that has Medicaid?” **Inpatient facility policies:** “We can have a patient who clearly needs inpatient care, but then they have no insurance or they’re from out of the country and then we can’t find an inpatient facility to accept them. So that’s a logistics barrier.” **Controversy around emergency detention policies:** “For people who are having imminent thoughts of hurting themselves, we are forced to hold them for safety like they can’t be allowed to leave, which I think is controversial and kind of a barrier to caring for them just because I think it angers people and feels like they’re getting their rights taken away.”	**Suicide screening policies:** “You might have patients that come in that are medical patients and you ask [suicide screening] questions, come to find out there’s more going on. So. we do well in the sense that we ask those questions regardless of why the patient is presenting to the ER.” **Triage/stratification by risk level:** “I know we have a policy regarding, depending on the levels of risk and what determines what needs to be done dependent on the level of risk.” **Policies in place to protect safety:** “Our structural support in the ED would be security and the monitored rooms and the availability of sitters.” **Community resource guide:** “Our hospital has a nice resource guide that they’ve created and I think it’s now posted on our website... It has emergency contacts, clothing assistance, housing, rent, a local food pantry, veterans assistance, mental health, education, pregnancy.” **Emergency detention policies:** “So the emergency detention order is when the patient has clearly marked that they are a harm to themselves... before to be able to keep someone against their will, I had to get signed documentation from the judge saying you can do this… back in July there was a law that came out that said, “Hey, if you feel like this person is unsafe to themselves or to others, and you feel like if you let them go really bad things are going to happen to themselves or to society or to their family, you have the ability now to make this call without calling the judge.” So, I have the ability for the next 48 hours to keep this patient until I feel like they are safe or until I get them to a safe place.”

**Subtheme:** Communication Structures	

**Barriers:** Gaps in communication	**Facilitators:** Established communication channels
**Lack of communication structures:** “I think there’s system barriers, for example, the fact that I have no way to call or speak to the behavioral health provider impacts our ability to implement things because I can’t discuss anything with them.” **Lack of knowledge about behavioral health resources:** “I’ve had colleagues go, “What do you put for resources?” and you’re like, I have no idea. I think we’re lagging behind on providing the best care because unfortunately I don’t think we know all the information. The loop of communication isn’t going around as, as easily as it should.”	**Collaboration with behavioral health already in place:** “Our social workers are always available to give them resources in addition to seeing our behavioral health team like the psychiatrist. Those are really the two big ones, two big services that I think we offer.” **Communication infrastructure for consultations already in place:** “I think [social workers] take care of the entire hospital, but they are just a phone call away and are very responsive to helping us when we need them.”

*ED*, emergency department.

**Table 3:** Implementation climate: perceived rewards, support, and expectations related to suicidality management.

**Subtheme:** Scope of Services	

**Barriers:** Suicidality management seen as out of scope	**Facilitators:** Suicidality management as an organizational priority
**Focus on stabilization vs treatment:** “You know, we aren’t involved in any long-term medications. We’re more in terms of stabilizing the patient, you know using more short-term medications to address the acute crisis.” **Lack of specialized services in the ED:** “The problem with the ER is that we’re like that jack of all trades with the master of none, like we have little pieces of everything, and I think there’s a lot of training we could use.” **Focus on other conditions in the ED:** “Behavioral health patients are one subset of our many patients, we have patients who are victims of violence, patients who have complicated medical needs, transplant patients, cancer patients, patients with life threatening issues like stroke, myocardial infarction, so those patients or those diagnoses tend to get a lot of resources and effort targeted at them.” **Competing demands/suicidality management is not prioritized:** “I feel like it’s valued, but not a lot. It’s valued in the sense that they don’t want someone to go home who was just seen in the hospital and commit suicide, because that would be bad press and bad for us from that standpoint. But it’s also not like this is a priority.” **Limited reimbursement:** “I think there’s probably not adequate reimbursement, particularly in pediatrics for mental health diagnoses. I think that limits the system from making it a priority and realizing that we have increased behavioral health patients and directing more resources to that end.	**Past organizational history of implementing innovations for suicidality management:** “I think [my organization values suicide prevention] because we ask everybody that walks through the door. We are always equipped for it, if that makes sense. We’re asking the questions. We’re implementing the things we need to implement.” **Prioritization of safety:** “Our manager is always on board with prioritizing safety and patient experience and just trying to make sure that people leave feeling cared for.”

**Subtheme:** Behavioral Health Saliency	

**Barriers:** Limited awareness of suicidality management	**Facilitators:** Awareness of suicidality management
**Not taking suicidal ideation seriously:** “When I worked inpatient psych they used to send people up there all the time. We would send people up there for even small things like if they claimed suicidal ideation once they would be sent up there. And however, I sometimes don’t feel like we take it as seriously down in our ED.” **Minimal presence of social work in the ED:** “Social workers have made themselves known recently. Unfortunately, I don’t feel like from the Emergency Department we utilize social work as much [as another ED]. I didn’t even know who our social work team was, probably until about a month ago.” **Lack of knowledge about prescription medications:** “What are my safe options, and can I be educated on the top risks and what medications I should prescribe it with, so that way I can at least bridge them until they get into primary care or even a psychiatric provider?” **Lack of knowledge about resources for referral / follow-up:** “A lot is who do we refer these people to because we don’t know. Behavioral health will evaluate, and they’ll say they’ll put resources in their discharge paperwork, but none of them gets relayed to us like we don’t have a list anywhere that we can send these people.”	**Presence of an organizational champion:** “Our certified nurse specialist has really made this a big push on asking those questions, so I do think there’s, they’re working on pushing it. There are people we can talk to, like, hey, I think this might help.” **Supports in place for behavioral health consults in the ED:** “We have [a behavioral health team], social workers, nurses, and psychiatrists available to consult.” **Supports in place for connecting patients with follow-up services:** “If someone wants to take the next step and sometimes if they do, social work will help them make the phone calls or, you know, help do an intake. It’s not like, here’s the resources, good luck. You know, somebody’s truly serious about that then you know we can help them kind of call around and make those phone calls and get what they need, and social work will help them with that.”

**Subtheme:** Leadership Support	

**Barriers:** Not supportive of suicidality management	**Facilitators:** Supportive of suicidality management
**Lack of leadership willingness to allocate resources towards behavioral health:** “I’m sure they’d be happy for us to do something if it didn’t cost them any money, but you know it depends on what we were asking for them for.” **Motivation of leaders not viewed as intrinsic:** “It’s more of a thing that they have to do because they get audited. I don’t know if it’s really something that’s a huge priority in their goals... I don’t think they really necessarily take into account like the therapeutic side of suicidal patients.”	**Leadership open/receptive to making improvements**: “I think they would be supportive of what can we do to improve and get better and make things better for the nursing staff and the patients too. And the, and the docs. I’m sure the docs would be willing to, to as well.” **Leadership support for suicide prevention/management:** “I know it’s something [another nurse] has already worked on in the past. It’s just like the safety of our patients and mental health crises and having suicidal thoughts. I think she’d be open to it and probably have a lot of wisdom on it just because it’s something she already has experienced with.” **Support across multiple organizational levels:** “It’s a collaborative thing. I know our medical director of medical affairs, he’s also an ER physician. He’s willing to have those discussions. What can we do to streamline a new workflow? What does everybody need? What kind of training do we need? So yeah, they’re pretty open to any concerns... Same as the manager.” **Leadership goals of protecting legal/reputational interests:** “It’s valued in the sense that they don’t want someone to go home who was just seen in the hospital and commit suicide, because that would be bad press and bad for us from that standpoint.”

*ED*, emergency department.

**Table 4:** Innovation values-fit: perceptions of how suicidality management as an innovation fulfills individual values.

**Subtheme:** Individual Values	

**Barriers:** Satisfied with current processes	**Facilitators:** Desire for better processes
**Belief that the ED is already providing the best care possible:** “I think we do the best that we can in getting them the right, you know, providing them that or sending them into like the mental health area.” **Personal beliefs about scope of ED services:** “I don’t think we have the ability to give them that, you know, a lot of that is more of a PCP thing on getting a patient on anxiety meds or depression meds or whatever it may be, and we don’t have the capacity for the follow up. So, it’s hard to make the emergency room an area where that can be handled because I feel like mental health medications or care required continued follow up and that’s not going to happen in the emergency room.” **Belief that change is needed beyond organization:** “There’s just a cultural change that needs to happen, not just ERs, society as a whole on how we can help treat mental health care. Unfortunately, I think it’s, more relies on a PCP, and sometimes people don’t have a PCP. So how do you fix that? Like I think there’s a bigger component to it than just the emergency room because we can only do so much in an emergency room.”	**Personal desire for more resources/better process for emergency detentions:** “Why don’t we have more options… a lot of these people I feel like are super angry and scarred and horribly mad at us when we decide to do [detain them], and sometimes it’s not because we decided to take their rights away, but now they’ve agreed to go somewhere, but we’re sending them four hours away from their family. They will never come back to us again if something were to happen because it didn’t go as smoothly as it could have. So, I think if we had these resources, we were able to provide them, not only would we be able to help prevent any things from going forward for them, prevent them from harming themselves or their families, but we would also get their trust back if they were to have another mental health crisis because the process just isn’t smooth at the moment.” **Personal desire to set patients up with additional resources for follow-up care:** “It’s kind of difficult because when we get repeat offenders, we kind of just hand them a sheet and it’s like okay, go because I mean, at some point, you gotta want to help yourself. However, with some of those people, I think it would be beneficial to kind of set them up with like offers and transportation and an appointment and send them that way rather than just kind of hand them a sheet of paper.”

**Subtheme:** Individual Attitudes	

**Barriers:** Stigmatizing views about suicidality management	**Facilitators:** Intrinsic value of suicidality management
**Beliefs that the ED is being taken advantage of:** “There’s some people that abuse the system when it comes to the emergency room and it makes it hard to identify who truly needs help.” **Beliefs that resources are being wasted:** “It’s just overly, which I guess a screening test is supposed to be that way, over inclusive. You don’t want to miss anybody who’s even potentially thinking of hurting themselves, but it is truly overinclusive. So, a lot of resources are spent on people who are just sad or upset or angry” **Beliefs about patient vs clinician responsibilities:** “I think what they’re doing is probably enough. I just think it’s up to the patient. And if they do get discharged, it’s up to the patient to follow up and it’s up to us to give them appropriate follow up.” **Stigma around seeking mental health services:** “I just wish there was a little bit more help, and I wish that it was more advertised that this is something people deal with because I think in a lot of ways it’s stigmatized very much... I feel like we could put up something for mental health to make our patients more willing and open to talk to us.”	**Recognition of impact of suicide screening:** “I had a patient who came in who was there for ankle pain or some sort of thing, and I was asking the ASQ questions and he admitted to me that he had thoughts of recently harming himself and ended up needing a sitter like we would have never found that out and been able to get those resources... he just wouldn’t have gotten the care he needed. However, he was able to do that because of those ASQ questions. When I first saw the questions, I was like, oh, these are very straightforward and might throw a lot of people off, but when I had that happen, I was like oh, these are like actually really important questions. I think these are great and I do appreciate them being straightforward.” **Recognition of potential service impact/beliefs that services could save lives:** “Save our patients lives, get them help before they ever get to that point. People, I think after COVID just really things started or, I don’t know maybe it’s always been that people are struggling but maybe it’s now more acceptable for you to talk about it. There’s a lot of people dealing with a lot of stuff mentally at home, at work, and if we can, whether it’s providing them resources of “Hey, I can get you an appointment here. It’s someone we have affiliation with. They can get you an appointment next week. Call them. Get scheduled.” Or here’s a hotline that you would call if you’re feeling this way in the meantime. Or you know what, here is more options.”

*ASQ, Ask* Suicide-Screening Questions toolkit; ED, emergency department; *PCP*, primary care physician.
